# Transferability and interpretability of the sepsis prediction models in the intensive care unit

**DOI:** 10.1186/s12911-022-02090-3

**Published:** 2022-12-29

**Authors:** Qiyu Chen, Ranran Li, ChihChe Lin, Chiming Lai, Dechang Chen, Hongping Qu, Yaling Huang, Wenlian Lu, Yaoqing Tang, Lei Li

**Affiliations:** 1 Department of Applied Mathematics, School of Mathematical Sciences, Fudan University, Shanghai, 200433 China; 2 Department of Critical Care Medicine, Ruijin Hospital, Shanghai Jiaotong University School of Medicine, Shanghai, 200025 China; 3 Shanghai Electric Group Co., Ltd., Central Academe, Shanghai, China

**Keywords:** Sepsis, Intensive care unit, Machine learning, Transfer learning, Prognostication, Model interpretability

## Abstract

**Background:**

We aimed to develop an early warning system for real-time sepsis prediction in the ICU by machine learning methods, with tools for interpretative analysis of the predictions. In particular, we focus on the deployment of the system in a target medical center with small historical samples.

**Methods:**

Light Gradient Boosting Machine (LightGBM) and multilayer perceptron (MLP) were trained on Medical Information Mart for Intensive Care (MIMIC-III) dataset and then finetuned on the private Historical Database of local Ruijin Hospital (HDRJH) using transfer learning technique. The Shapley Additive Explanations (SHAP) analysis was employed to characterize the feature importance in the prediction inference. Ultimately, the performance of the sepsis prediction system was further evaluated in the real-world study in the ICU of the target Ruijin Hospital.

**Results:**

The datasets comprised 6891 patients from MIMIC-III, 453 from HDRJH, and 67 from Ruijin real-world data. The area under the receiver operating characteristic curves (AUCs) for LightGBM and MLP models derived from MIMIC-III were 0.98 − 0.98 and 0.95 − 0.96 respectively on MIMIC-III dataset, and, in comparison, 0.82 − 0.86 and 0.84 − 0.87 respectively on HDRJH, from 1 to  5 h preceding. After transfer learning and ensemble learning, the AUCs of the final ensemble model were enhanced to 0.94 − 0.94 on HDRJH and to 0.86 − 0.9 in the real-world study in the ICU of the target Ruijin Hospital. In addition, the SHAP analysis illustrated the importance of age, antibiotics, net balance, and ventilation for sepsis prediction, making the model interpretable.

**Conclusions:**

Our machine learning model allows accurate real-time prediction of sepsis within 5-h preceding. Transfer learning can effectively improve the feasibility to deploy the prediction model in the target cohort, and ameliorate the model performance for external validation. SHAP analysis indicates that the role of antibiotic usage and fluid management needs further investigation. We argue that our system and methodology have the potential to improve ICU management by helping medical practitioners identify at-sepsis-risk patients and prepare for timely diagnosis and intervention.

*Trial registration*: NCT05088850 (retrospectively registered).

**Supplementary Information:**

The online version contains supplementary material available at 10.1186/s12911-022-02090-3.

## Background

Sepsis, an infection-induced syndrome of physiological, pathological, and biochemical abnormalities, is a global healthcare issue associated with unacceptably high mortality and long-term morbidity among patients in the intensive care unit (ICU) [1, 2], and is responsible for a substantial cost burden on health care resources [3]. Early detection and timely administration of appropriate antibiotics are important for improving the prognosis and survival of septic patients [4]. However, nonspecific symptoms of sepsis may cause delayed diagnosis and intervention, leading to the high mortality of septic patients [5].

Machine learning has emerged as a promising tool for the early detection of sepsis occurrence based on electronic medical records, laboratory data, and biomedical signals [6–14]. Several prospective studies have shown that the implementation of machine learning-based sepsis prediction algorithms can reduce in-hospital mortality and length of stay [15, 16]. Except for the excellent prediction performance, the translation of these risk prediction models into clinical practice requires external independent validation to determine the generalizability of the model to different cohorts [17]. However, most of the newly proposed risk prediction models have worsened performance when applied to external samples [18]. Re-training of the prediction model on local datasets in the target medical center might enhance the predictive accuracy in the specific situation [19]. Based on the same objective, transfer learning has been reported to improve model performance when the dataset is small in the target medical center [20, 21]. Additionally, the interpretability of machine learning models reflects the extent to which the decision-making process of the model can be understood and accepted in clinical practice. The lack of interpretability for most available prediction models is currently the major barrier to their clinical adoption [22, 23]. The objective of this study is to develop an interpretable early warning system (named SEPRES, Sepsis PREdiction System) for real-time sepsis prediction in the ICU and to improve its generalizability to the target medical center through the transfer learning technique.

## Methods

SEPRES includes a data integration system equipped with a sepsis early warning module. The data integration system collects, stores, processes, and displays medical data. The sepsis early warning module included a sepsis prediction model and an interpretative tool. The sepsis prediction model is an ensemble of multiple machine learning models. The interpretative tool provides information on how the model works by assigning importance to the input features. Our study complies with the relevant reporting guidelines, namely the Transparent Reporting of a multivariable prediction model for Individual Prognosis Or Diagnosis (TRIPOD) statement [24].

### Data acquisition

#### Data sources

Our study used the Medical Information Mart for Intensive Care (MIMIC-III) database (version 1.4) [25] and the private Historical Database of Ruijin Hospital (HDRJH). MIMIC-III encompasses 61,532 patients admitted to the ICU at Beth Israel Deaconess Medical Center in Boston from 2001 to 2012, and HDRJH encompasses approximately 1777 patients from 2011 to 2019. In addition to retrospective data, we also collected predictions of consecutive 67 patients from the SEPRES system running in the ICU at Ruijin (RJ) Hospital between February 2021 and June 2021 as a validation of the model in the real world.

#### Sepsis definitions

We defined sepsis according to the definition of the Third International Consensus for sepsis (Sepsis-3) [2], combining suspected infection and Sequential Organ Failure Assessment (SOFA) score. Details can be found in Additional file 1: Appendix 1.

#### Feature extraction

We extracted 78 and 63 patient variables from the MIMIC-III and HDRJH, respectively. After data cleaning, we extracted these variables as features, i.e., maximum, average, median, and minimum, at hourly intervals, and the missing data were padded by the nearest value before or a preset default value. We filtered out 1057 positive and 5834 negative patients in the MIMIC-III dataset, and 144 positive and 309 negative patients in the HDRJH dataset, respectively. We used a 5-h time window from the patients to predict sepsis. See Additional file 1: Appendix 1 for details.

### Machine learning models

In the following two sections, we describe the methodology for developing a sepsis prediction model that outputs the risk of sepsis onset within 5-h preceding at most. To improve the prediction performance in the specific hospital and to avoid the poor performance of direct training due to its insufficient data, the models were first trained in MIMIC-III and then finetuned in HDRJH using transfer learning techniques. The ultimate sepsis prediction model was obtained by integrating multiple models using ensemble learning techniques.

Multiple models were trained on the MIMIC-III dataset, including support vector machine (SVM), multilayer perceptron (MLP), gradient boosting machine (GBM), and long short-term memory (LSTM). For GBM, we used XGBoost [26] and LightGBM [27] as implementations.

We utilized the standard training methods to train these models with necessary normalization which can be summarized by the following formula:

$$\widetilde{{x_{j}^{i} }} = \frac{{x_{j}^{i} - \mathop {\min }\limits_{{i^{\prime}}} x_{j}^{{i^{\prime}}} }}{{\mathop {\max }\limits_{{i^{\prime}}} x_{j}^{{i^{\prime}}} - \mathop {\min }\limits_{{i^{\prime}}} x_{j}^{{i^{\prime}}} }}$$

where ${x}_{j}^{i}$ is the value of the j-th feature of the i-th sample, and $\widetilde{{x}_{j}^{i}}$ is the value after scalarization. The hyperparameters and structures of each model were tuned based on the validation set.

See Additional file 1: Appendixs 4 and 5 for details.

### Transfer learning

Based on the integrated considerations of the performance including accuracy, the area under the receiver operating characteristic curve (AUC), sensitivity, and inference speed on the MIMIC-III dataset, we selected the LightGBM and MLP for sepsis prediction on the RJ Hospital data.

The process of transfer learning can be divided into two steps. First, we normalized the data in the MIMIC-III dataset and the HDRJH dataset respectively so that the features were all scaled between 0 and 1. Second, we performed the transfer of the model parameters. Specifically, for LightGBM, the previous four hours of features in MIMIC-III were masked during training to assist transfer learning. After that, inputs from the HDRJH dataset were fed to finetune with the initial parameter values taken from the trained model from MIMIC-III. For MLP, we first froze the parameters of the first three of the six layers of the MIMIC-III models and initialize the parameters of the last three layers. After training on HDRJH, the models were unfrozen and fine-tuning is performed. We also used an ensemble learning method to integrate the LightGBM and MLP models by taking the inference average. The ensemble model is employed for practical sepsis prediction in RJ Hospital.

### Interpretive analysis

We interpreted our prediction models using Shapley additive explanation (SHAP) [28], a game theory-based approach that assigns an importance value to each feature of each prediction.

### Real-time prediction system

We detail the implementation of our real-time prediction system in [29]. When a model inference command is executed, the sepsis early warning module obtains real-time features of the patient from the data integration system via SQL query statements, which are then preprocessed, inferred, and interpreted by the module. The data integration system includes a physical server with the PostgreSQL database for storage of sepsis warning-related data and a webserver deploying the portal for user access. The medical device integration hub was placed at the bedside, receiving and transmitting data to the data integration system with a time delay of less than 10 s. Using the network or RS-232 interface, the data integration system can integrate data from IntelliVue Information Center, ventilators, Philips ICCA system, Laboratory Information System (LIS), and Hospital Information System (HIS).

## Results

### Characteristics of patients from different datasets

The baselines of characteristics of patients from MIMIC-III, HDRJH, and Ruijin real-world data were analyzed. As shown in Additional file 1: Table S2, the baselines of most characteristics were significantly different between patients from MIMIC-III and patients from HDRJH except for 21 characteristics. Therefore, to avoid the influence of these differences on model performance in the target cohort, retraining of the model was performed on HDRJH using the transfer learning technique after training on MIMIC-III.

### Prediction performance on internal and external validation of MIMIC-III

The performance of our sepsis prediction models has been evaluated based on the accuracy, AUC, sensitivity, and specificity on the test set. The default classification threshold is 0.5. As shown in Additional file 1: Fig. S3, the GBM-based models (XGBoost and LightGBM) outperformed others (See Additional file 1: Table S3 for the performance of the five models). Furthermore, we compared our LightGBM and MLP models with other models that were developed from MIMIC-III, using Sepsis-3 criteria and reporting the prediction outcomes within 5 h before the onset of sepsis. As shown in Table 1, our LightGBM and MLP models were superior to the others, with AUC of 0.98 and 0.96 respectively. However, it should be noticed that although these models all used MIMIC-III database, there were still differences in the training and test sets due to specific data extraction and sepsis criteria.

**Table 1:** The results of different models on the MIMIC-III dataset

Model	Preceding hours	Accuracy	AUC	Sensitivity	Specificity
InSight	4	0.57	0.74	0.8	0.54
AISE	4	0.64	0.84	0.85	0.64
MGP-TCN	4	–	Approximately 0.85	–	–
DTW-KNN	4	–	Approximately 0.88	–	–
MLA	0	–	0.88	0.8	0.78
MLA	24	–	0.84	0.8	0.72
DSPA	4	–	0.98	–	–
MGP-AttTCN	4	–	0.75	–	–
NAVOY Sepsis	3	0.81	0.84	0.74	0.83
LightGBM	4	0.91	0.98	0.85	0.97
MLP	4	0.85	0.96	0.73	0.96

*AISE* Artificial Intelligence Sepsis Expert, *MGP-TCN* Multi-task Gaussian Process and Temporal Convolutional Networks, *DTW-KNN* Dynamic Time Warping and K-Nearest Neighbours, *MLA* Machine Learning Algorithm, *DSPA* Deep SOFA-Sepsis Prediction Algorithm, *MGP-AttTCN* Multi-task Gaussian Process and Attention Time Convolutional Network

For the external validation of our LightGBM and MLP models, we evaluated their prediction performances on HDRJH, the dataset from the target medical center (the ICU in RJ Hospital). The AUCs were 0.82 − 0.86 and 0.84 − 0.87 respectively on HDRJH from 1 − 5 h preceding, indicating the substantially worsened performance of these models when applied to external independent cohorts.

### Improved prediction performance on HDRJH after transfer learning

To improve the prediction performance on HDRJH, these models were retrained and ensembled using transfer learning and ensemble learning technique. As shown in Table 2, transfer learning improved the prediction performance when deploying the models derived from the public dataset (MIMIC-III) to the target hospital (HDRJH). The ultimate AUCs of the ensemble sepsis prediction model were 0.94 − 0.94 from 1 to 5 h preceding on HDRJH, as shown in Additional file 1: Table S4.

**Table 2:** The results of models trained on different datasets on the HDRJH test set

Model	Transfer learning	Training set	Accuracy	AUC
LightGBM	N	MIMIC-III	0.74	0.86
LightGBM	N	HDRJH	0.81	0.92
LightGBM	Y	MIMIC-III + HDRJH	0.83	0.93
MLP	N	MIMIC-III	0.76	0.86
MLP	N	HDRJH	0.84	0.92
MLP	Y	MIMIC-III + HDRJH	0.78	0.93

These models predict sepsis in 4-h preceding and the complete results can be found in Additional file 1: Tables S5 and S6. MIMIC-III + HDRJH means training on MIMIC-III first and then tuning on the training set of HDRJH by transfer learning techniques

**Fig. 1:**
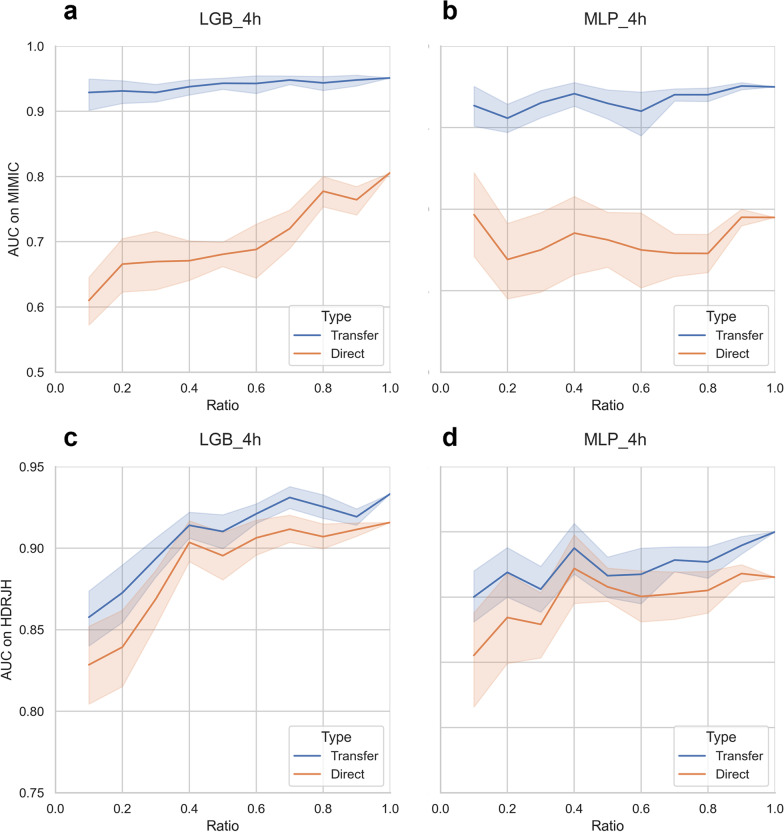
The results of models trained on sampled HDRJH dataset. The training set of HDRJH was sampled at different ratios to simulate a medical center with fewer records. These trained models were tested on the common MIMIC-III test set (top) and the HDRJH test set (bottom), respectively. The shaded part in the figure represents the 95% confidence interval. These models predict sepsis in 4-h preceding and the complete results can be found in Additional file 1: Figs. S5–S8

Furthermore, as shown in Fig. 1, LightGBM and MLP models showed consistent transfer benefits in the target hospital at different sampling ratios of the target hospital dataset. Meanwhile, the models after transfer learning showed higher AUCs on MIMIC-III, indicating improved generalizability of the model to different datasets.

### Feature interpretability of the prediction models

**Fig. 2:**
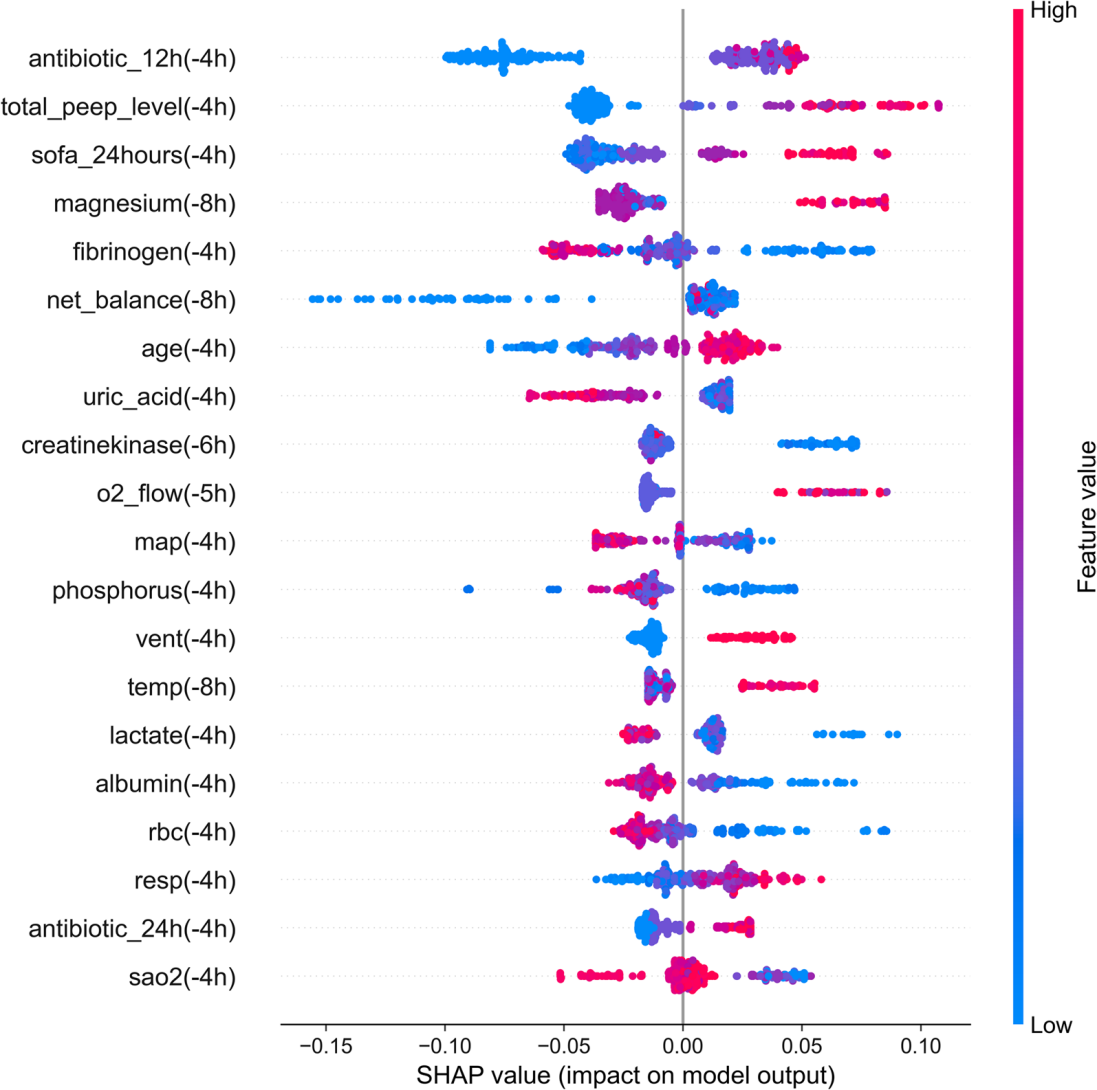
Feature importance of LightGBM model in the sepsis early warning module. The model predicted sepsis in 4-h preceding and the complete results can be found in Additional file 1: Fig. S10

The top 20 features for the LightGBM model predicting sepsis in 4-h preceding were shown in Fig. 2, and the results for full analysis of LightGBM and MLP models were shown in Additional file 1: Fig. S10, S11. Some of these features (antibiotics, respiratory rate, temperature, ventilation, and heart rate) were related to the definition of Sepsis-3 or Systemic Inflammatory Response Syndrome (SIRS). Additionally, the association of some of these features (respiratory rate [30], fibrinogen [31], net balance [32], and age [33]) with the severity or mortality of sepsis has been reported. These data indicate the good interpretability of our prediction model for clinical application.

### Prediction performance in the real world

Each patient was labeled by the change in SOFA score and the doctor’s examination for infection. Data from the control group and near onset of sepsis in the case group were included in the analysis. As shown in Table 3, the AUCs for sepsis predictions in 1 − 5 h preceding were 0.86 − 0.90.

**Table 3:** The results of real-world data

Preceding hours	Accuracy	AUC	Sensitivity	Specificity
1	0.82	0.86	0.83	0.82
2	0.84	0.88	0.87	0.78
3	0.85	0.9	0.86	0.81
4	0.85	0.9	0.88	0.78
5	0.86	0.89	0.9	0.76

**Fig. 3:**
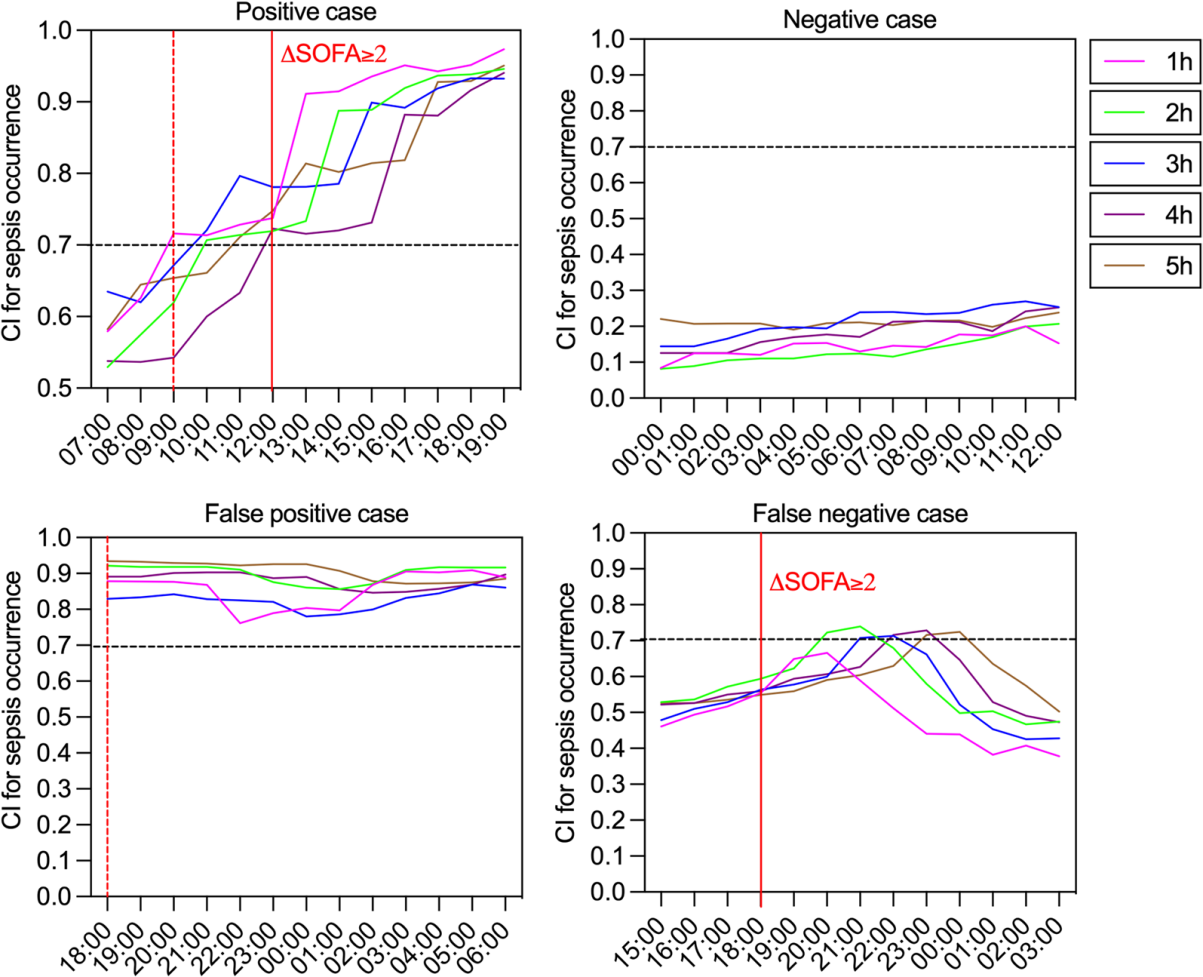
Some illustrative examples of prediction. Each subplot described the confidence index for multiple models (Y-axis) at the indicated time (X-axis). (i) The condition of the patient aggravated in the early morning, with multiple organ dysfunction, and the patient was diagnosed with sepsis at noon. Our model prediction exceeded the warning threshold of 0.7 for the prediction at 9:00 AM. (ii) Despite the high SOFA score (7.0), there was no evidence of ΔSOFA ≥ 2 within 72 h. Consistently, the predictions were all lower than the threshold. (iii) Although the patient’s SOFA score was stable at 6.0, our model made incorrect predictions of sepsis. (iv) The SOFA score showed an increase from 6.0 to 9.0 at 06:00 PM. In combination with evidence of infection, the patient was diagnosed with sepsis. However, the prediction was below the warning threshold

In the real-world study, the classification threshold was increased to 0.7 to reduce the false alarm rate of sepsis warnings. Figure 3 illustrated examples of the prediction of sepsis by SEPRES over a random period (See Additional file 1: Appendix 10 for more details). In the continuously early warning process of 67 patients admitted to the ICU, 22 septic patients and 29 non-septic patients were correctly predicted, whereas 17 non-septic patients and 6 septic patients were incorrectly predicted as false-positive and false-negative cases.

## Discussion

Machine learning has been considered a promising method for sepsis prediction in the ICU [6–16, 20–22]. Early diagnosis and timely management of septic patients can effectively improve the prognosis [34]. However, sepsis may not be diagnosed in time in the clinic due to the day-night shift and inattention of medical staff. Therefore, an accurate and efficient early prediction system for sepsis at the bedside is urgently needed. In this study, we established an ICU bedside sepsis early warning system, SEPRES, to conduct real-time sepsis prediction for patients in the ICU by integrating IntelliVue Information Center, ventilators, Philips ICCA system, LIS, and HIS data. Although SEPRES could not provide a definitive basis for our therapeutic regime, the predicted probability of sepsis occurrence allows us to pay more attention to at-sepsis-risk patients.

Generalizability is the major obstacle to the deployment of machine learning into medical practice. Sufficiently large data size is crucial for the training of the machine learning model to achieve good performance. Moreover, the performance of the model derived from one cohort is always worsened when applied to external independent cohorts due to the differences in race, medical environment, disease type, and disease severity in different cohorts. In addition to this, as new tests and techniques are added, new features may help our prediction task, but direct inclusion into existing machine learning models is usually not feasible. In our study, we deployed the transfer learning technique to improve the performance of our models in the target medical center. The transfer learning process effectively improved the prediction AUCs of LightGBM and MLP models on the HDRJH dataset, learns patterns from additional features, and showed consistent benefits across different data sizes of the target cohort. Hence, we argue that transfer learning might be a promising and feasible strategy to maintain the effectiveness of the trans-center deployment of machine learning models. Furthermore, transfer learning has been applied to similar domains or similar tasks in several medical fields, reducing the size requirements of the target dataset, and improving the training speed and the prediction performance [35–38]. In our context, transfer learning can be used to predict different types of diseases, such as disseminated intravascular coagulation (DIC) or acute kidney injury (AKI).

Moreover, the lack of interpretability of these data-driven models prevents the practitioners to trust and accept these machine learning models in the clinic. In the present study, SHAP analysis as the interpretive tool helps medical practitioners identify top risk factors. What needs to be noted is fluid net balance and the use of antibiotics. Due to the difficulty of collecting the net balance data in most datasets, net balance has neither been considered as a feature for most machine learning models based on MIMIC-III datasets nor been analyzed as an important factor for sepsis prediction inference. Indeed, the positive cumulative fluid balance has been reported to be an independent predictor of ICU mortality [32]. Moreover, Lin et al. [39] have shown that patients with an early positive fluid balance have an increased risk of developing venous thromboembolism. Our SHAP analysis further emphasized the importance of careful fluid management in critically ill patients. In addition, Our SHAP analysis results suggested that heavy antibiotic use corresponds with an increased predictive value for the occurrence of sepsis. Due to the uncertainty regarding antibiotic initiation in patients with suspected infection, the use of antibiotics is mostly empirical in ICU patients [40]. It has been reported that inappropriate antibiotic treatment may accelerate the death of mice via increasing gut proliferation and systemic spreading of a multi-drug resistant (MDR) *Escherichia coli* strain [41]. Moreover, the initial inappropriate broad-spectrum antibiotic therapy may promote the dissemination of multidrug-resistant bacteria (MDRB), increase opportunistic infection, and is associated with poor prognoses of patients [42, 43]. The SHAP analysis inspired us to focus more on antibiotic use and fluid management, but it should be emphasized that this evidence is not sufficient for intervention in clinical practice, but should be judged based on the patient's clinical features.

SEPRES has certain limitations. First, we enrolled only patients who were non-septic during the entire period in the ICU as negative controls. The enrollment condition may be too pure which may cause false-positive cases. Second, as we observed in consecutive case studies, patients diagnosed with sepsis shortly after being transferred to the ICU were difficult to be predicted by our model, which is probably due to that our model tends to give lower predictions when the collected data are limited. Finally, variables such as antibiotics and mechanical ventilation were incorporated into our model, resulting in the influence of the model predictions by the subjective behavior of the doctor. However, considering that the use of antibiotics and mechanical ventilation are associated with the severity of the patient, it is essential to include them in our model. These limitations will be addressed in future work through diverse methods, including fine-grained labeling, inclusion of data collected from the data integration system in the future, and methods to enhance generalization capabilities such as data augmentation or feature selection. We also highlight that the application of transfer learning and interpretive tools can significantly improve the generalization and interpretability of the model but still possesses a distance to totally solve it.

## Conclusions

In conclusion, the early prediction of sepsis occurrence by our SEPRES has the potential to guide medical practitioners to appropriately pay more attention to at-sepsis-risk patients, leading to early diagnosis of sepsis and more efficient ICU patient management. Our SHAP analysis suggests the need for further investigation regarding the role of fluid net balance and the use of antibiotics. Moreover, with the help of the data integration system to collect necessary features and data, the workflow of SEPRES can be applied to disease warnings other than sepsis in the ICU, such as DIC and AKI. Furthermore, our work confirms the effectiveness of applying transfer learning in sepsis prediction, improving the predictive ability and reducing the number of records required in the target medical center. The proposed system can be applied to a larger number of medical centers with a certain number of records through transfer learning.

## Supplementary Information

**Additional file 1:** Supplementary material.

## Data Availability

The MIMIC-III datasets generated and/or analyzed during the current study are available in the MIMIC repository, https://mimic.physionet.org/. The HDRJH dataset and RJ real-world data were approved for limited use by the Ruijin Hospital Ethics Committee, and were not publicly available. The code of the model inference was uploaded into GitHub under “SEPRES”.

## Abbreviations

ICU: Intensive care unit
SEPRES: SEpsis PREdiction system
TRIPOD: Transparent reporting of a multivariable prediction model for individual prognosis or diagnosis
MIMIC-III: Medical information mart for intensive care
HDRJH: Historical database of Ruijin Hospital
RJ: Ruijin
SOFA: Sequential organ failure assessment
SVM: Support vector machine
MLP: Multilayer perceptron
GBM: Gradient boosting machine
LSTM: Long short-term memory
AUC: Area under the receiver operating characteristic curve
SHAP: Shapley additive explanation
LIS: Laboratory information system
HIS: Hospital information system
AISE: Artificial intelligence sepsis expert
MGP-TCN: Multi-task Gaussian process and temporal convolutional networks
DTW-KNN: Dynamic time warping and K-Nearest neighbours
MLA: Machine learning algorithm
DSPA: Deep SOFA-Sepsis prediction algorithm
MGP-AttTCN: Multi-task Gaussian process and attention time convolutional network
SIRS: Systemic inflammatory response syndrome
DIC: Disseminated intravascular coagulation
AKI: Acute kidney injury
MDR: Multi-drug resistant
MDRB: Multidrug-resistant bacteria
TDM: Therapeutic drug monitoring
